# Radiocarbon Flux Measurements Provide Insight into Why a Pyroligneous Acid Product Stimulates Plant Growth

**DOI:** 10.3390/ijms25084207

**Published:** 2024-04-10

**Authors:** Randi Noel, Michael J. Schueller, Richard A. Ferrieri

**Affiliations:** 1 Missouri Research Reactor Center, University of Missouri, Columbia, MO 65211, USA; rlnhmh@umsystem.edu (R.N.); schuellerm@missouri.edu (M.J.S.); 2 Division of Plant Science & Technology, University of Missouri, Columbia, MO 65211, USA; 3 Chemistry Department, University of Missouri, Columbia, MO 65211, USA; 4 Interdisciplinary Plant Group, University of Missouri, Columbia, MO 65211, USA

**Keywords:** pyroligneous acid, Coriphol™, chlorophyll, beta-carotene, radiocarbon flux analysis, carbon-11

## Abstract

Agriculture in the 21st century faces many formidable challenges with the growing global population. Increasing demands on the planet’s natural resources already tax existing agricultural practices. Today, many farmers are using biochemical treatments to improve their yields. Commercialized organic biostimulants exist in the form of pyroligneous acid generated by burning agricultural waste products. Recently, we examined the mechanisms through which a commercial pyroligneous acid product, Coriphol™, manufactured by Corigin Solutions, Inc., stimulates plant growth. During the 2023 growing season, outdoor studies were conducted in soybean to examine the effects of different Coriphol™ treatment concentrations on plant growth. Plant height, number of leaves, and leaf size were positively impacted in a dose-dependent manner with 2 gallon/acre soil treatments being optimal. At harvest, this level of treatment boosted crop yield by 40%. To gain an understanding of why Coriphol™ improves plant fitness, follow-up laboratory-based studies were conducted using radiocarbon flux analysis. Here, radioactive ^11^CO_2_ was administered to live plants and comparisons were made between untreated soybean plants and plants treated at an equivalent Coriphol™ dose of 2 gallons/acre. Leaf metabolites were analyzed using radio-high-performance liquid chromatography for [^11^C]-chlorophyll (*Chl*) *a* and *b* components, as well as [^11^C]-β-carotene (β-*Car*) where fractional yields were used to calculate metabolic rates of synthesis. Altogether, Coriphol™ treatment boosted rates of *Chl a*, *Chl b*, and β-*Car* biosynthesis 3-fold, 2.6-fold, and 4.7-fold, respectively, and also increased their metabolic turnover 2.2-fold, 2.1-fold, and 3.9-fold, respectively. Also, the *Chl a/b* ratio increased from 3.1 to 3.4 with treatment. Altogether, these effects contributed to a 13.8% increase in leaf carbon capture.

## 1. Introduction

Agriculture in the 21st century faces many formidable challenges, with the growing and economically empowered global population placing increasing demands on the planet’s natural resources and energy supplies. The increased demand for food and energy to feed this growing population and to heat and provide electricity for their homes is already taxing existing agricultural practices and natural resources. Projections indicate that feeding a world population of 9.77 billion people by 2050 [1], coupled with an improved diet, will require further increases in food production of more than 60 percent [2]. Production in developing countries will need to almost double. Furthermore, as we attempt to increase reliance on a bioenergy-based market, agriculture will need to increase outputs of biomass and chemical feedstocks to keep future energy and industrial resources in balance. These efforts will likely increase agricultural waste products that are presently under-utilized.

Pyroligneous acid, wood vinegar, and liquid smoke are all names synonymous with products that are made from the condensation of vapors produced during the high-temperature oxygen-free pyrolysis of agricultural and forestry biomass [3,4,5,6]. These products typically are a reddish-brown aqueous liquid with a distinctive smokey smell which can vary in terms of phenols, carboxylic acids, and other light water-soluble oxygenated compounds of different solubilities depending on the biomass feedstock used in the process [7].

In recent years, aspects of this technology have become commercialized and are sold as biostimulants for promoting plant growth. For example, Corigin Solutions, Inc. (Merced, CA, USA) manufactures Coriphol™ and markets the product as a liquid organic plant growth enhancer for improving crop yields and crop quality when applied to the field. Their product contains more than 150 chemical components (Supplementary Table S1), including organic acids, phenols, alcohols, aldehydes, esters, ethers, and ketones. The overall organic content of Coriphol™ reaches 15% by weight, with the rest being water. Such organic content is high with respect to typical wood vinegar/liquid smoke products. Moreover, phenols, suspected to be the main active agents for the biostimulant effect, represent a large fraction of the organic content on an acid-free basis.

Despite the positive feedback for crop performance with improvements in yield and quality reported for tomato, rapeseed, cotton, lettuce, and onion [8,9,10,11,12,13,14], producers of pyroligneous acid products that are marketed as biostimulants for plant growth know very little about the how their products work. The objectives of the present work were two-fold. The first objective was to examine the performance of one commercial product, Coriphol™, at different applied doses to the soil to determine the optimal dose needed for growth stimulation in a single crop model: soybean. Soybean was selected as the model because the product had not yet been tested for its performance on this crop and because soybean is a major crop with approximately 87.5 million acres planted annually in the United States. The second objective of this work was to identify key metabolic features underpinning plant growth using radiocarbon flux analysis to understand how this product stimulates growth at an optimal dose. In the long term, we feel that such information will help to broaden the use of such products in future farming practices in the fight against global hunger.

## 2. Results

### 2.1. Outdoor Plant Growth Performance

**Figure 1:**
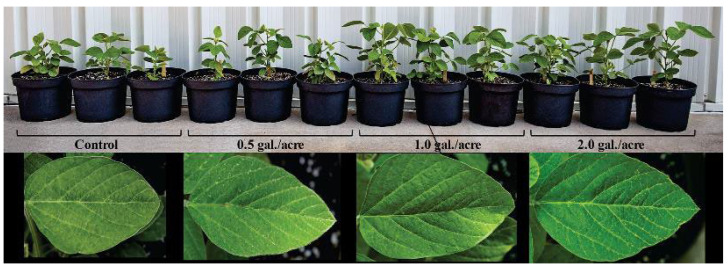
Digital photos of a random sampling of soybean plants taken in week 5 for untreated control plants and plants treated at germination with doses of Coriphol™ equivalent to 0.5, 1.0, and 2.0 gal./acre. Leaf photos were taken of fully expanded leaves from the 2nd trifoliate. Each leaf photo frame was scaled to 11 cm.

**Figure 2:**
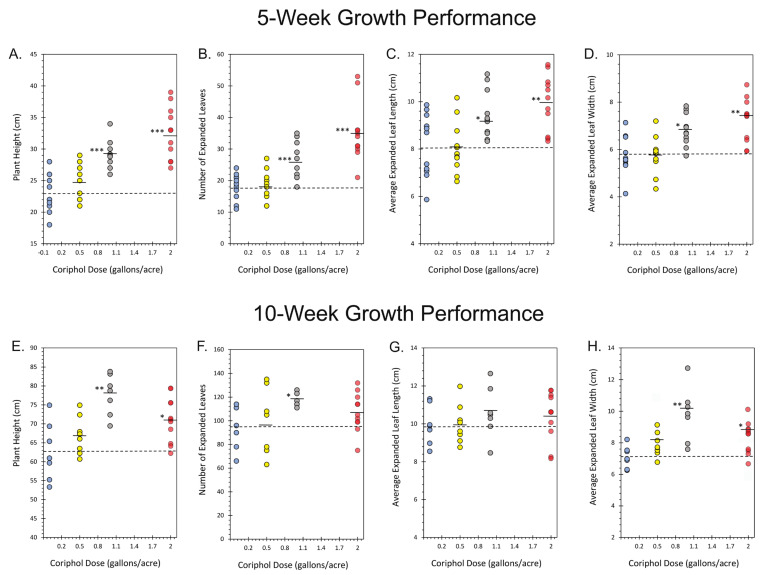
Plant growth metrics, including plant height (Panels (**A**,**E**)), number of leaves (Panels (**B**,**F**)), leaf length (Panels (**C**,**G**)), and leaf width (Panels (**D**,**H**)) were acquired in week 5 (Panels (**A**–**D**)) and again in week 10 (Panels (**E**–**H**)). Data points from individual plants (N = 9–10) are shown for untreated control plants and plants treated with 0.5, 1.0, and 2.0 gal./acre Coriphol™. Dashed lines in each panel reflect the mean values of the control plant. Solid bars in each panel reflect mean values for Coriphol™-treated plants. Levels of significance are depicted as follows: * *p* < 0.05; ** *p* < 0.01; *** *p* < 0.001.

In week 5, a representative sampling of plants (N = 3 from each treatment cohort) was lined up and photographed (Figure 1) representing untreated control plants and plants treated at germination with doses of Coriphol™ equivalent to 0.5, 1.0, and 2.0 gallons per acre (gal./acre). Leaf photos were taken of fully expanded leaves from the second trifoliate. Growth performance metrics, including plant height, leaf number, and leaf size, were also acquired using a tape measure and ruler. Measurements were taken in week 5 and again in week 10 (Figure 2).

Results in week 5 showed a clear dose dependency with treatment where plant height increased from 23.05 ± 0.91 to 32.55 ± 1.25 cm for untreated and 2.0 gal./acre treatment, respectively. Leaf count increased from 18.18 ± 1.24 to 35.18 ± 2.81 for untreated and 2.0 gal./acre treatment, respectively. Leaf length measured from the tip to the petiole connection increased from 8.17 ± 0.40 to 9.97 ± 0.36 cm for untreated and 2.0 gal./acre treatment, respectively, and leaf width increased from 5.83 ± 0.25 to 7.23 ± 0.28 for untreated and 2.0 gal./acre treatment, respectively. Results in week 10 showed a similar pattern of behavior, although plants treated with 1.0 gal./acre Coriphol™ seemed to outperform those treated at the higher dose.

### 2.2. Outdoor Harvest Data

**Figure 3:**
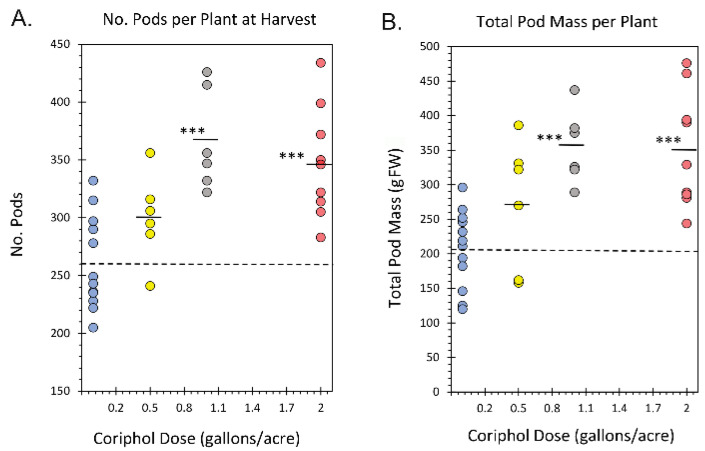
Soybean crop yield data at week 20 harvest presented by the number of pods picked per plant (Panel (**A**)) and the total mass of pods per plant (Panel (**B**)) in grams fresh weight (gFW). Blue data points reflect untreated control plants, yellow data points reflect plants treated with 0.5 gal./acre Coriphol™, grey data points reflect plants treated with 1.0 gal./acre Coriphol™, and red data points reflect plants treated with 2.0 gal./acre Coriphol™. Level of significance is depicted by ***, which represents *p* < 0.001.

In week 20, soybean pods were picked, tallied, and weighed for a determination of crop yield (Figure 3). Pods were selected for harvest if they showed signs of developing beans inside (some pods were not fully developed at the time while most pods were mature). The number of pods per plant increased from 260.83 ± 11.64 pods in untreated plants to 300.69 ± 15.42 pods with 0.5 gal./acre Coriphol™ treatments, 366.33 ± 17.85 pods with 1.0 gal./acre treatments, and 347.22 ± 16.02 pods with 2.0 gal/acre treatments. Like growth performance traits in week 10, plants treated at 1.0 gal./acre slightly outperformed those treated at 2.0 gal/acre in the number of pods harvested, although this difference was not statistically significant. Total pod mass also increased from 207.25 ± 16.12 gFW for untreated plants to 271.50 ± 38.33 gFW with 0.5 gal./acre treatments, 355.17 ± 21.71 gFW with 1.0 gal./acre treatments, and 350.05 ± 27.81 gFW with 2.0 gal./acre treatments.

**Figure 4:**
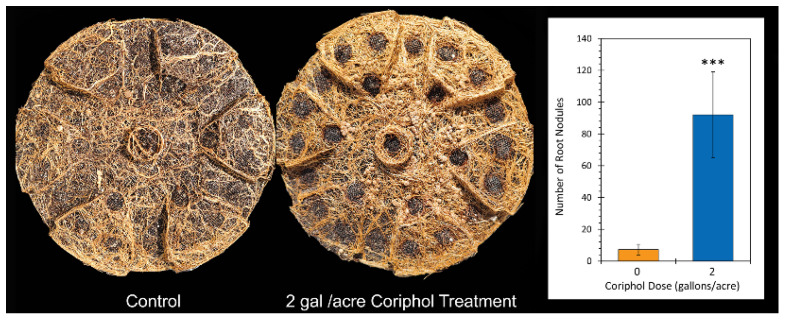
The left-side pictures show a representative set of photographs taken of the roots along the bottom of the planting pot for an untreated control plant and a plant treated at 2 gal./acre with Coriphol™. Mean values ± SE for number of nodules tallied across a sample of *n* = 6 plants in each cohort are shown by the bars in the adjacent graph. Level of significance is depicted by ***, which represents *p* < 0.001.

After harvest and during the deconstruction of the outdoor growth project, we observed that there was a higher root density at the bottom layer with a greater number of root nodules in the Coriphol™-treated plants than in the untreated plants. The extent of nodulation was surprising since non-inoculated seed was used during sowing. Digital photographs were taken of the bottom portion of the root mass (Figure 4) for *n* = 6 untreated plants and an equivalent number of plants treated with 2.0 gal./acre Coriphol™. The number of root nodules was tallied from each photograph and averaged. When tallied, the root nodule count was observed to rise from 17.25 ± 3.35 nodules in untreated plants to 92.0 ± 27.14 nodules in plants treated at 2.0 gal./acre with Coriphol™.

### 2.3. Laboratory Radiocarbon Flux Measurements

After the 2023 growing season, studies were moved back into the laboratory to examine mechanisms for enhanced plant growth with Coriphol™ treatment. Here, plants were grown in smaller 6-inch pots under controlled environmental conditions (see Section 4) and subjected to radiocarbon flux analysis using ^11^CO_2_ at the V4 stage of development.

**Figure 5:**
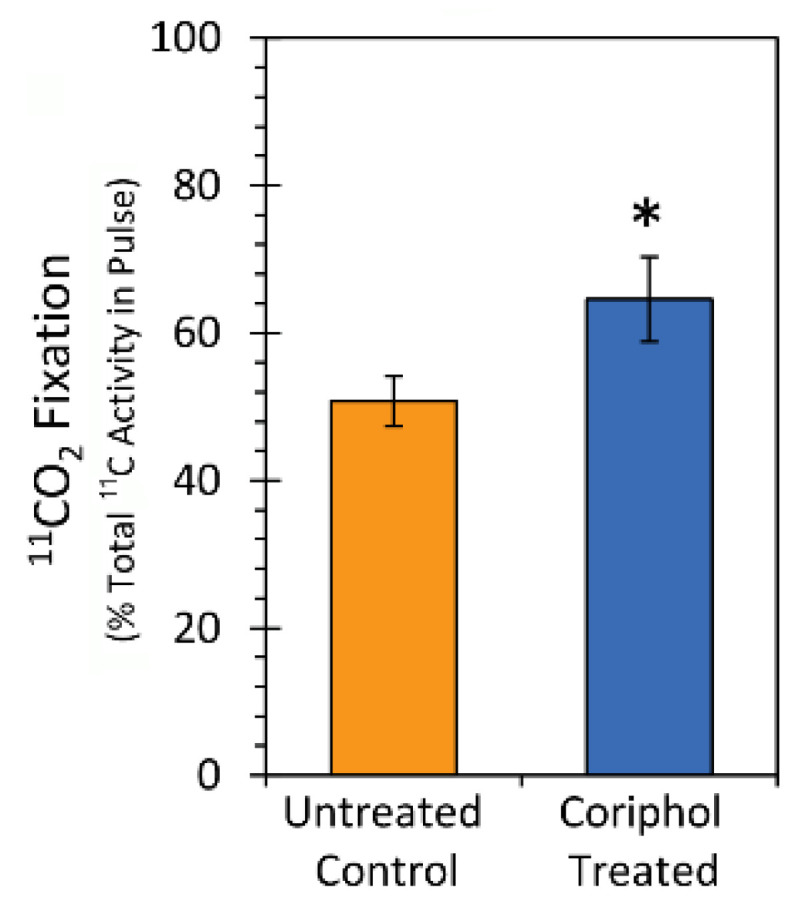
Relative percent ^11^CO_2_ fixation based on radioactivity delivered in the pulse. Data were normalized to a fixed leaf tissue mass affixed within the leaf cell. Data bars reflect mean values ± SE for *n* = 6 replicates in each treatment type. Level of significance is depicted by *, which represents *p* < 0.05.

A positive aspect to administering the ^11^CO_2_ tracer as a discrete pulse in air is that we were able to measure leaf carbon fixation. Data shown in Figure 5 reflect the relative ^11^CO_2_ fixation based on the amount of radioactivity delivered in each pulse. All data were normalized to a fixed mass of leaf tissue affixed within the leaf cell. All pulses were conducted at a fixed cell illumination of 450 µmol m^−2^ s^−1^ in red-blue light (equal intensity). Results showed that ^11^CO_2_ fixation significantly increased from 50.76 ± 3.39% to 64.57 ± 5.76% with 2.0 gal./acre Coriphol™ treatment resulting in a net 13.8% increase in leaf carbon capture.

**Figure 6:**
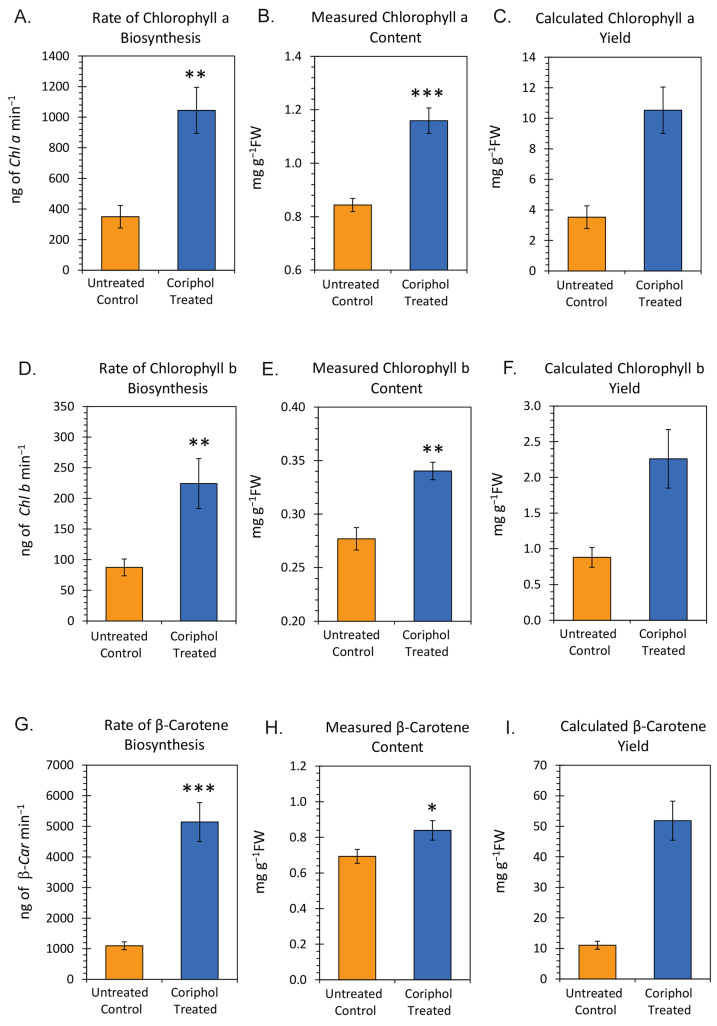
Rates of *Chl a*, *Chl b*, and β-*Car* biosynthesis in nanograms per minute (ng min^−1^) are shown in Panels (**A**,**D**,**G**), respectively. The amounts of endogenous *Chl a*, *Chl b*, and β-*Car* in milligrams per gram fresh weight leaf tissue (mg g^−1^FW) are shown in Panels (**B**,**E**,**H**), respectively. The rates of biosynthesis were applied to the lifetime of the leaf tissue to arrive at calculated amounts of *Chl a*, *Chl b*, and β-*Car* in mg g^−1^FW shown in Panels (**C**,**F**,**I**). All data bars reflect mean values ± SE for *n* = 6 replicates in each treatment type. Levels of significance are depicted by * *p* < 0.05; ** *p* < 0.01; *** *p* < 0.001.

After 20 min of incubation with a radiotracer, leaf tissue was collected and radiometabolites were extracted and analyzed by radio–UV high-performance liquid chromatography (see Section 4). Fractional ^11^C-yields for *Chl a*, *Chl b*, and β-*Car* were converted to rates of biosynthesis in nanograms of metabolite per minute (ng min^−1^) as described in Section 4. Mean values ± SE were shown in the data bars of Figure 6, as well as Panels A, D, and G for *Chl a*, *Chl b*, and β-*Car*, respectively. Results showed that the rate for *Chl a* biosynthesis increased from 349.53 ± 73.72 ng min^−1^ to 1044.51 ± 150.46 ng min^−1^ with treatment culminating in a 3-fold increase in the rate. The rate for *Chl b* biosynthesis increased from 87.20 ± 13.71 ng min^−1^ to 224.22 ± 40.73 ng min^−1^ with treatment culminating in a 2.6-fold increase in the rate. Finally, the rate for β-*Car* biosynthesis increased from 1097.38 ± 129.22 ng min^−1^ to 5143.23 ± 633.44 ng min^−1^ with treatment culminating in a 4.7-fold increase in the rate.

Panels B, E, and H in Figure 6 reflect the amounts of endogenous *Chl a*, *Chl b*, and β-*Car* that were measured by UV absorption in each extracted tissue sample analyzed. Results here showed that the content of *Chl a* increased from 0.84 ± 0.02 mg g^−1^FW to 1.16 ± 0.05 mg g^−1^FW with treatment. The content of *Chl b* increased from 0.28 ± 0.01 mg g^−1^FW to 0.34 ± 0.01 mg g^−1^FW with treatment. Finally, the content of β-*Car* increased from 0.69 ± 0.04 mg g^−1^FW to 0.84 ± 0.05 mg g^−1^FW with treatment.

Panels C, F, and I in Figure 6 reflect the amounts of endogenous *Chl a*, *Chl b*, and β-*Car* that were calculated based on the measured rates of biosynthesis integrated over the lifetime of the leaf. Results here showed that the calculated content of *Chl a* increased from 3.52 ± 0.74 mg g^−1^FW to 10.53 ± 1.52 mg g^−1^FW with treatment. The calculated content of *Chl b* increased from 0.88 ± 0.14 mg g^−1^FW to 2.26 ± 0.41 mg g^−1^FW with treatment. Finally, the calculated content of β-*Car* increased from 11.06 ± 1.33 mg g^−1^FW to 51.84 ± 6.39 mg g^−1^FW with treatment.

Using data on actual measured amounts of *Chl a*, *Chl b*, and β-*Car* and their calculated amounts based on their respective rates of formation, we were able to examine the influence of Coriphol™ treatment on individual pigment metabolism. Table 1 summarizes the results from these calculations.

We note that calculated pigment content based on biosynthetic rates was always higher than the actual measured values (Ratio Calc./Act.) for each pigment examined suggesting that all these substrates undergo some level of metabolism. Interestingly, the ratio Calc./Act. for β-*Car* was significantly higher than that of the chlorophylls, suggesting that its metabolic turnover is much higher than that of the chlorophylls. The comparison of pigment metabolic turnover between untreated control plants and Coriphol™-treated plants (depicted as the fold change in metabolic turnover Table 1) showed that both *Chl a* and *Chl b* exhibited nearly identical behavior with 2.17 ± 0.57-fold and 2.11 ± 0.52-fold increases in metabolic turnover, respectively, with treatment. However, β-*Car* exhibited a significantly higher response to treatment with a 3.85 ± 0.74-fold increase.

**Figure 7:**
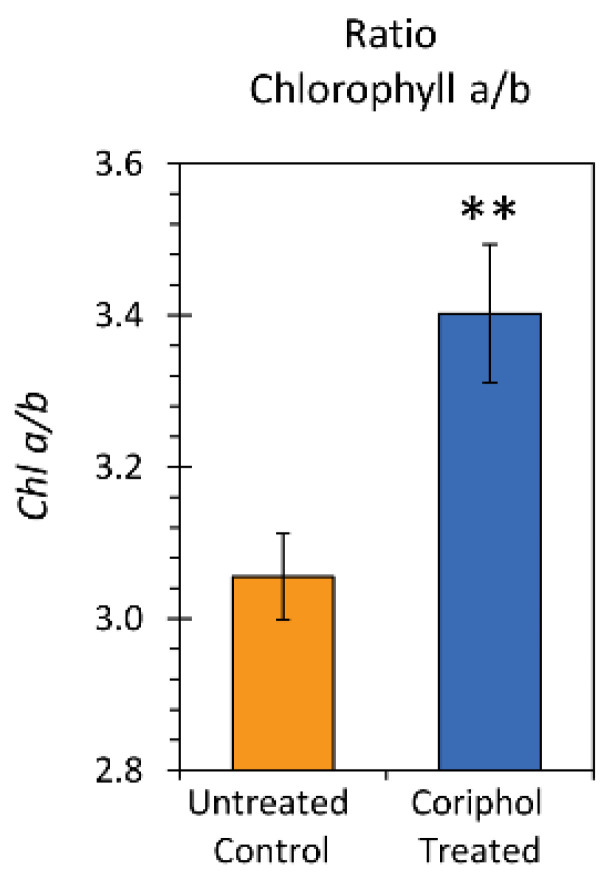
Ratio *Chl a/b* for untreated control plants and plants treated with Coriphol™ at an equivalent dose of 2.0 gal/acre. Data bars reflect mean values ± SE for *n* = 6 replicates in each cohort. Level of significance is depicted by **, which represents *p* < 0.01.

Because *Chl a/b* can be an important metric for assessing leaf nitrogen content, we plotted this ratio (Figure 7) for untreated control plants and plants treated with Coriphol™ at an equivalent dose of 2.0 gal./acre. Results show that *Chl a/b* increased from 3.06 ± 0.06 in untreated plants to 3.40 ± 0.09 in treated plants.

## 3. Discussion

**Figure 8:**
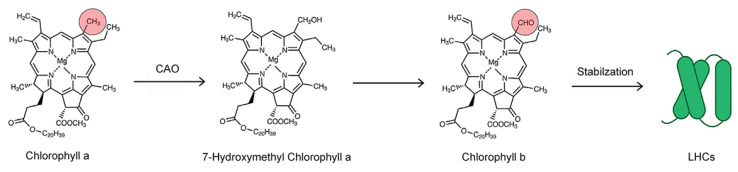
Structures, pathways, and modes of action for *Chl a* and *Chl b*.

Green algae, terrestrial plants, and some cyanobacteria utilize two types of chlorophyll (*Chl a* and *Chl b*) for their photosynthesis [15]. The structure for *Chl a* possesses a methyl group in the C7 position (Figure 8), while that for *Chl b* has a formyl group in the same position. *Chl a* and *Chl b* have distinct absorption spectra in the blue and red regions, which enable this combination of pigments to absorb wider ranges of light spectra [16].

Years ago, several biosynthetic pathways for *Chl b* synthesis were proposed [17] prior to the actual identification of the pathway. Now, it is accepted that *Chl b* is synthesized from *Chl a* by the oxidation of the methyl group in the C7 position. This process proceeds through 7-hydroxymethyl chlorophyll *a* by the action of the chlorophyllide *a* oxygenase (CAO) enzyme [18].

Solar energy is mostly captured by a light-harvesting chlorophyll protein complex (LHC) of the photosynthetic apparatus. In higher plants, both *Chl a* and *Chl b* are bound to the LHC complex [19,20]. However, *Chl b* is essential for the complexe’s functionality [21,22] because its binding stabilizes protein in the thylakoid membranes [23,24], allowing for the efficient transfer of light energy to *Chl a* [25].

In a past study [26], over 800 soybean genotypes were screened for their chlorophyll *a/b* ratio where 93% had *Chl a/b* ratios greater than 3.05. It is well established that the irradiance of plants will modulate leaf anatomy and leaf physiology [27,28,29]. In high-irradiance environments, leaves become thicker and possess an increased mesophyll-to-surface area. Typically, in high light conditions, the total chlorophyll content per unit leaf area will decrease while the chlorophyll *a/b* ratio will increase compared to that of low light conditions [27,28,29,30]. Due to its predictable response to irradiance, *Chl a/b* has been proposed as a bioassay to assess the irradiance of a plant or its ability to tolerate high light stress [31].

Additionally, the synthesis of the photosynthetic apparatus requires large amounts of N where the proportion of leaf N allocated to the chloroplast is approximately 75% [32]. Significant correlations between leaf photosynthesis and leaf N content have been documented for many species, including soybean [33,34,35,36,37,38], and a positive correlation between leaf N and chlorophyll content is also well documented [38,39,40,41,42,43,44,45,46]. Past studies have also shown that the *Chl a/b* ratio can serve as an indicator of the protein makeup within a chloroplast and hence N partitioning within the leaf [47,48,49] based on the positive relationship of *Chl a/b* with the ratio of the PSII cores to the LHC.

In the present work, we note that the *Chl a/b* ratio of untreated control plants matched that of past published work [26]. Our observation that this ratio rose to 3.4 with Coriphol™ treatment suggests that leaf nitrogen content was higher than that of untreated plants, although we did not measure this level. However, we also noted that the roots of treated plants had a significantly higher amount of root nodules suggesting that biological nitrogen fixation from the rhizobia would be higher, providing an enhancement to plant nitrogen uptake.

During photosynthesis, carotenoids normally serve as antenna pigments. Carotenoids increase the efficiency of photosynthesis by absorbing blue-green light and transferring this energy to chlorophyll. They are bound to antenna protein complexes that channel energy to the photosystem II (PSII) reaction centers where the first energy storing electron transfer events take place. Thus, wherever there is chlorophyll there are carotenoids [50,51,52]. An important role in the photosynthetic process has to do with the ability of certain carotenoids to quench chlorophyll triplets, ^3^P_680,_ thus preventing their production of singlet molecular oxygen (^1^O_2_) that can be highly reactive and can be extremely toxic to the plant cell by causing oxidative damage [53]. However, despite the presence of β-*Car* in the isolated PSII reaction centers, past spectroscopy studies have shown its yield was less than 3% while that of the triplet chlorophyll was over 30% [54,55]. Hence, the role of β-*Car* in the photosynthetic process is not to quench chlorophyll triplets in the PSII reaction centers preventing them from forming ^1^O_2_, but rather it directly quenches ^1^O_2_ preventing oxidative damage [50,51,56].

In the past, it has been noted that at high light intensities, once the rate of photosynthetic electron transport reaches a maximum, there is a gradual intensity-dependent increase in the yield of carotenoid triplets. This behavior is referred to as the valve reaction [57] and reflects the fact that, once the photochemical reactions become saturated, the yield of chlorophyll triplets increases and hence more quenching by carotenoids occurs. We see this behavior in the present work in that while Coriphol™ treatment boosted β-*Car* biosynthesis, its metabolic turnover was increased 3.85-fold with treatment as a compensatory action to mitigate oxidative damage promoted by the 13.8% increase in leaf carbon capture driven by increased photosynthesis. Of course, if left unchecked, this increase in the photosynthetic process would saturate the electron transfer reactions in the PSII cores and eventually lead to photoinhibition and the loss of photosynthetic activity.

## 4. Materials and Methods

### 4.1. Outdoor Plant Growth

For outdoor studies, soybean seeds (MorSoy variety 4812E, MFA Inc., Columbia, MO, USA) were sown into a 2.7-gallon pot filled with ProMix (Premier Tech Horticulture, Inc., Salt Lake City, UT, USA). A capful of fertilizer (~1.2 g) containing nitrogen, phosphate, and potash (14-14-14, Osmocote, Smart-Release Plant Food Flower & Vegetable., The Scotts Company, Marysville, OH, USA) was mixed into the ProMix before sowing. Fertilizer was reapplied to pots 30 days after germination and again 60 days later. Pots were placed on elevated tables outside and were connected to drip irrigation lines providing water twice daily in for a daily total of 2 L of water per pot. Four cohorts of plants were grown in replicate including untreated control plants and plants were treated with 100 mL doses of Coriphol™ equivalent to 0.5, 1.0, and 2.0 gal./acre. Treatments were applied to the ProMix at germination and reapplied after 5- and 10-weeks of growth. Pot locations were rotated every 3 weeks to eliminate biases in outdoor growth conditions.

Growth performance was measured in week 5 and again in week 10. Using a measuring stick, plant height was measured from the soil of the pot up to the highest point of the plant in centimeters. The number of leaves was tabulated at each session. And, finally, the upper most fully expanded source leaf was targeted for measurements of leaf length and width.

### 4.2. Plant Growth for Laboratory Radiotracer Studies

Soybean seeds (MorSoy variety 4812E, MFA Inc., Columbia, MO, USA) were germinated in 6-inch pots filled with ProMix mixed with 0.5 g Osmocote™ fertilizer (ICL, Inc., St. Louis, MO, USA). Pots were placed in a commercial growth chamber (model PGC-15, Percival Scientific, Inc., Perry, IA, USA) initially covered in clear plastic until seedling germinated at which time the plastic was removed. The growth chamber was set to operate under a 12 h photoperiod at 500 µmol m^−2^ s^−1^ light intensity, and temperatures of 25 °C/20 °C (light/dark) with relative humidity at 60%. Plants were studied using ^11^CO_2_ at the V4 stage.

### 4.3. Production and Administration of Radioactive ^11^CO_2_

**Figure 9:**
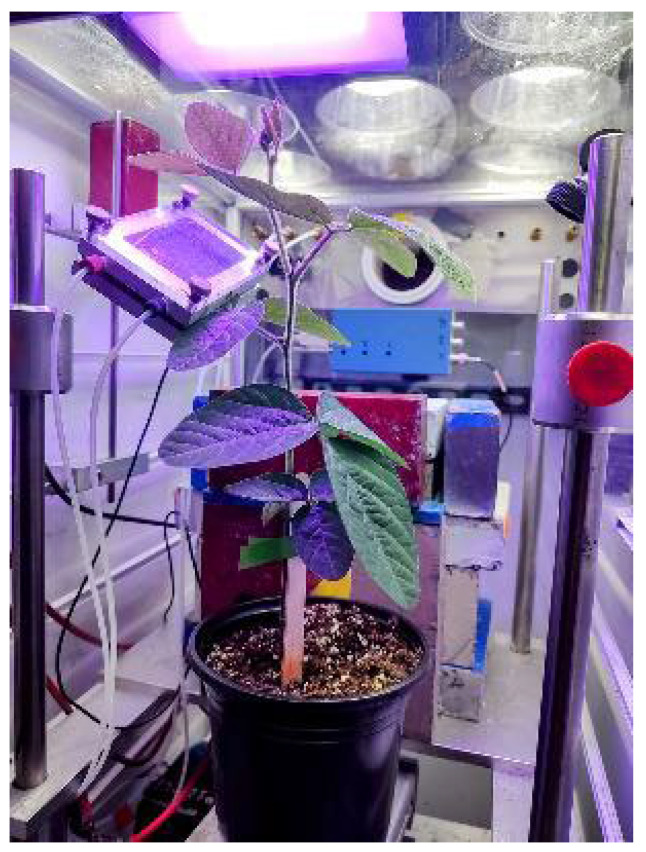
Photograph depicting the plant set up with the leaf cell affixed to an intact leaf and the system ready to receive a pulse of ^11^CO_2_.

^11^CO_2_ (t_½_ 20.4 min) was produced on the GE PETtrace Cyclotron (GE HealthCare, Chicago, IL, USA) located at the University of Missouri Research Reactor Center using high-pressure research-grade N_2_ gas target irradiated with a 16.4 MeV proton beam to generate ^11^C via the ^14^N(p,α)^11^C nuclear transformation [58,59]. The ^11^CO_2_ was trapped on molecular sieve 4 Å at ambient temperature, then desorbed at 350 °C, quickly releasing it into an air stream at 200 mL min^−1^ as a discrete pulse for labeling a leaf affixed within a 5 × 10 cm lighted (500 µmol m^−2^ s^−1^) leaf cell to ensure a steady level of fixation (Figure 9).

For continuity, tracer was administered a 2nd fully expanded trifoliate leaf. This “load” leaf was affixed within the cell and was pulse-fed ^11^CO_2_ for 1 min, then chased with normal air for the duration of exposure [60]. A PIN diode radiation detector (Carroll Ramsey Associates, Berkeley, CA, USA) attached to the bottom of the leaf cell enabled continuous measurement of radioactivity levels within the cell during the initial pulse and in the minutes directly following to give information on ^11^CO_2_ fixation. The “load” leaf was incubated for 20 min after which exposed tissue was harvested for radiolabeled pigment analysis.

### 4.4. Leaf Pigment Analysis

Approximately 500 mg of fresh, exposed leaf tissue, weighed to within 1 mg accuracy was fresh-ground in 3 mL cold buffer (0.2 M Tris-HCl, pH 8.0 in acetone, *v*/*v*) using 200 mg of 60–80 mesh silica powder (Ashland Inc., Irving, TX, USA) as an abrasive. The buffer was removed by pipette and a 40 µL aliquot subjected to pigment assay using radio–UV high-performance liquid chromatography (HPLC) as described below. Three additional extractions were performed on the tissue pellet using 3 mL aqueous acetone to remove remaining trace soluble radiolabeled metabolites. Aliquots from each of the washings were measured on a gamma counter. The washed pellet was dried for 20 min at 80 °C also measured on the gamma counter. After radioactive decay and analytical fraction corrections were applied, the data were summed to yield a total ^11^C-activity fixed by the leaf section. This value was later used to calculate fraction yields of each of the radiolabeled leaf pigments targeted in the HPLC analysis as described below.

This analysis was carried out using HPLC (Sonntek, Inc., Upper Saddle River, NJ, USA) using a 250 mm x 4.6 mm inner diameter Phenomenex C18 Hypersil column (Phenomenex Corp., Torrance, CA, USA) heated to 30 °C and a linear-gradient-programed mobile-phase system set at a flowrate of 1.2 mL min^−1^ and comprising 100% solvent A (1 M ammonium acetate: methanol, 20:80, *v*/*v*), programmed to 100% solvent B, (acetone–methanol, 20:80 *v*/*v*) over 15 min, and held at 100% solvent B for an additional 10 min. The system was interfaced with a SmartLine 2500 variable wavelength UV detector (Knauer, Inc., Berlin, Germany) set to 445 nm coupled with a NaI gamma radiation detector (Eckert and Ziegler, Inc., Wilmington, MA, USA) enabling direct measurement of the ^12^C-unlabelled and ^11^C-labelled chlorophylls and β-carotene as they eluted the column. Data for both UV absorption and radioactivity were acquired simultaneously using PeakSimple™ chromatography software v4.88 (SRI, Inc., Torrance, CA, USA). UV absorption data were compared to standard calibration curves constructed for the individual chlorophylls and β-carotene providing absolute amounts for these metabolites in units of nanogram (ng) per gram fresh weight (g^−1^FW) tissue. Radioactive metabolite peaks for [^11^C]-chlorophyll *a*, [^11^C]-chlorophyll *b*, and [^11^C]-β-carotene were quantified using the same software, corrected for radioactive decay, and corrected for differences in efficiencies between the gamma counter and the HPLC’s NaI detector to generate fraction yields of each ^11^C-pigment based on total ^11^C-radioactivity acquired by the plant.

### 4.5. Calculation of Leaf Pigment Metabolic Turnover

Each discrete pulse of ^11^CO_2_ to the plant leaf was in a stream of air containing an ambient concentration of ^12^CO_2_ (417 ppm). Each pulse was of 1 min duration. Using the ^11^CO_2_ fixation value that was measured from the radiation detector embedded in the leaf cell, we calculated the amount of ^12^C in nanograms that was fixed at the same time as ^11^C. Next, we multiplied the ^11^C-fractional yields of each pigment, as measured above, by the mass of ^12^C that was fixed during the 1 min pulse to arrive at biosynthesis rates in nanogram per minute (ng min^−1^).

### 4.6. Statistical Analysis

Data were subjected to analysis of variance (ANOVA) using SigmaPlot version 14.5. Tukey’s HSD test was used for post hoc correction of comparisons of treatments to untreated control plants. Significance was set at a level of *p* < 0.05.

## 5. Conclusions

The present work clearly demonstrated that the treatment of soil with the commercial pyroligneous acid biostimulant, Coriphol™, at optimal doses equivalent to 1 to 2 gal./acre enhanced soybean plant growth and improved yield. Furthermore, this work showed that such treatment increased leaf carbon capture by 13.8%. This increase in the photosynthetic process was driven by our observed increase in the biosynthetic rates and amounts of leaf chlorophylls, as well as that of the important pigment, β-*Car* where the later plays an important role in mitigating oxidative stress, a consequence of increased photosynthesis. What we do not know currently is whether the increase in leaf chlorophylls is a direct action of treatment with pyroligneous acid or a consequence of other upstream actions. For example, manganese (Mn) is an essential micronutrient for plant growth [61,62] where it is a cofactor for the oxygen-evolving complex of the photosynthetic process. Here, Mn sparks the process by splitting water after the PSII cores harness sunlight to initiate the conversion of CO_2_ and water into sugar and molecular oxygen [63]. From our past work using radioactive ^52^Mn^2+^, we showed that certain root-associating bacteria in maize will promote host uptake of Mn with increased translocation to leaves, improving the photosynthetic process as evidenced by increased ^11^CO_2_ fixation and increased leaf chlorophyll content [64]. We also showed that the same results could be achieved simply by enriching the Mn content in the growth media.

Natural soil Mn levels range between 450 and 500 mg kg^−1^DW, while plant tissues possess approximately 220 mg Mn kg^−1^DW of tissue [65]. Furthermore, a recent study showed that concentrated pyroligneous acid possesses only about 4 mg Mn L^−1^. When diluted for field application, this level of Mn reflects a tiny fraction of the natural Mn abundance in soil, and therefore treatment will have no effect on altering that level. However, the question remains whether certain organic substrates present within pyroligneous acid products can facilitate the sequestering and plant uptake of soil Mn, beneficially affecting chlorophyll biosynthesis. Our past studies have shown that certain polyphenolics present in a pyroligneous acid product in high abundance, namely resorcinol (1,2-dihyroxybenzene) and catechol (1,3-dihydroxybenzene) can alter vascular connectivity within live plants [66], suggesting that these substances are actively taken up by the plant. The pyroligneous acid product, Coriphol™, which we examined in the present study had a 10.76% relative abundance of dihydroxybenzenes in the organic content (Supplementary Table S1). Many of these polyphenolic compounds are capable of complexing with metals [67,68], and especially with Mn [69], suggesting that these substrates could act as vehicles for conveying Mn into the plant. Indeed, past studies on *Azolla filiculoides* treated with pyroligneous acid showed significant increases in plant tissue Mn content, although no mechanism for this increase was offered [70]. In future studies, we plan to use radioactive ^52^Mn^2+^ to measure the uptake kinetics of the metal as a function of Coriphol™ treatment, which should shed additional light on the mechanisms of action.

## Figures and Tables

**Table 1:** Effect of Coriphol™ treatment on metabolic turnover of leaf pigments.

	Mean Actual Amount ^a^	Standard Error	Mean Calculated Amount ^b^	Standard Error	Ratio Calc./Actual	Propagated Error	Fold Change in Metabolic Turnover ^c^	Propagated Error
***Chl a***								
Untreated	0.84	0.02	3.52	0.74	4.19	0.89	2.17	0.57
Treated	1.16	0.05	10.53	1.52	9.08	1.37		
***Chl b***								
Untreated	0.28	0.01	0.88	0.14	3.14	0.51	2.11	0.52
Treated	0.34	0.01	2.26	0.41	6.65	1.22		
**β-*Car***								
Untreated	0.69	0.04	11.06	1.33	16.03	2.14	3.85	0.74
Treated	0.84	0.05	51.84	6.39	61.71	8.45		

^a^ Mean actual amounts (in mg g^−1^FW) were measured using the HPLC UV detector response. ^b^ Mean calculated amounts (in mg g^−1^FW) were calculated using the biosynthetic rates times the lifetime of the leaf. ^c^ Fold change in metabolic turnover calculated from the change in the Calc./Actual Ratio between untreated and treated plants.

## Data Availability

All data needed to evaluate the conclusions in the paper are present in the main text.

## Supplementary Materials

The following supporting information can be downloaded at https://www.mdpi.com/article/10.3390/ijms25084207/s1.
