# Supplementary material for: Radiocarbon Flux Measurements Provide Insight into Why a Pyroligneous Acid Product Stimulates Plant Growth
Source: Int J Mol Sci. 2024 Apr 10;25(8):4207. doi: 10.3390/ijms25084207 (PMC11050665; doi:10.3390/ijms25084207)
Supplement: Supplementary file 1 [file ijms-25-04207-s001.zip › ijms-2961308-supplementary.pdf]

Table S1. Relative Percent Distribution of Identified Organic Substrates in Coriphol™ Product

Compound	Rel. %	Compound	Rel. %	Compound	Rel. %	Compound	Rel. %
butanoic acid	0.07	ethanediol diacetate	0.07	dimethylcyclopentenone	0.65	aminomethyl pyridine oxide	0.06
cyclopentanone	0.12	dimethylcyclopentenone	0.04	methylfuranone	0.25	methyloctanone	0.05
pyruvic acid butyl ester	1.84	dihydromethylfuranone	0.37	ethylmethylbutyrate	0.29	cresol	1.56
methyl dioxolane	0.02	dimethyl pyridine	0.06	dimethyl pentanone	0.11	isocytosine	0.04
cyclopentenone	1.92	methylcyclopentenone	2.51	isopropylphenylpentenol	0.06	methyl heptenone	0.07
maleic acid anhydride	0.02	acetyloxybutanone	8.04	phenol	2.64	methyl furanone	0.06
methylpyridine	0.32	pyranone	0.02	propionyl ethylacetate	0.11	methyl hexenone	0.07
acetyloxypropanone	11.68	methylhexanol acetate	0.74	butoxybutanol	0.11	propanoic acid phenyl ester	0.03
dihydropyranone	0.08	propionylethylacetate	0.22	ethyl dimethylpyridine	0.01	cyclohexanediol	0.15
3-methylbutanol acetate	1.83	pentanoic acid oxo methylester	0.04	diisopropylidene acetone	0.06	dimethyl norbornanol	0.13
dimethylpyridine	0.04	hydroxymethyl butanone	0.22	3-ethylcyclopentenone	0.38	phenol	0.03
methylcyclopentenone	0.84	dimethylcyclopentenone	0.23	dihydrodimethylfuranone	0.14	dimethyl phenol	0.58
furanylethanone	1.40	furanol acetate	6.84	acetyloxy pentanone	1.38	isopropyl hexanone	0.06
hydroxybutanoic acid	0.01	dihydro dimethylfuranone	0.38	methyl pyrolidine dione	0.35	methyl propanoic acid anhydride	0.03
hydroxy methyl butanone	0.14	methylisobutyrate	0.25	dihydro methyl propylpyrazole	0.14	guaiacol	3.24
propanoic acid anhydride	0.01	oxobutanoic acid hexyl ester	0.16	dimethyl hexenone	0.05	octanolacetate	0.02
dimethylpyridine	0.14	methyl butanoate	0.13	methyl nitroimidazol	0.14	acetoxy-methyl-cyclopentenone	6.53
cyclohexenone	0.13	dimethoxycyclopentenone	0.01	dimethyl hydroxybutyric acid lactone	0.10	furan-2(methylthio)methyl	0.11
dimethylcyclopentenone	0.02	dimethoxycyclopentenone	0.12	hydroxy pyridine	0.19	pentanoic acid methyl-methylene isopropyl ester	0.77
hydroxymethylpentenone	0.15	hydroxy isobutyric acid acetate	0.09	butoxyethyl acetate	0.12	methoxy phenol	0.09
ethanediol diacetate	0.07	propanediol diacetate	0.10	acetyl dihydofuranone	0.02	acetoxy methylfuraldehyde	0.05

Compound	Rel. %	Compound	Rel. %	Compound	Rel. %	Compound	Rel. %
dimethyl cyclopentadione acetyl derivatives	0.19	methyl benzenediol	1.96	propenyl methoxyphenol	0.04	dimethoxy hydroxy benzaldehyde	0.04
ethyl thiophenol acetyl derivatives	0.04	trimethylbutyl pyrazine	0.05	dimethyl methoxy phenol	0.07	dimethoxy benzoic acid	0.01
acetyl dihydrofuranone	0.47	dimethyl ethyl thio benzene	0.09	methoxy acetophenone	0.09	propenyl dihydroxy benzene	0.02
ethyl hydroxy cyclopentenone	0.15	dimethoxy phenol	5.43	methoxy dimethylphenol	0.10	methoxy hydroxy benzoic acid	0.29
diacetyl	0.15	methyl benzenediol	3.01	dimethyl dihydroxy phenol	0.04	dihydroxy propenylbenzene	0.04
methoxyphenol	0.12	methyl benzenediol	0.02	methoxy benzene diol	0.02	hydroxy dimethoxy acetyl benzene	0.07
cyclohexene-ol dimethylacetate	1.42	methyl benzenediol	0.71	dimethoxy ethylphenol	0.66	dihydroxy phenyl propanone	0.02
ethylthiophenol	0.02	dimethyl benzenediol	0.21	dihydroxy methyl benzaldehyde	0.37	allyl dihydroxy methoxy benzene	0.01
methoxy methyl phenol	0.60	vanillin	0.02	dihydroxyacetrophenone	0.33	demethoxy isopropyloxy benzaldehyde	0.03
methyl dihydopyrandione	1.11	propyl benzene diol	0.04	benzoic acid methoxy methyl ester	0.02	dimethoxy hydroxy phenyl acetone	0.20
dihydroxybenzene	10.76	benzene thiol dimethylpropyl	0.05	dihydroxyacetophenone	0.03	dimethoxy hydroxy phenyl propanone	0.01
ethyl thiophenol	0.04	dimethylbenzene diol	0.49	isopropylbenzene thiol	0.05	trimethoxy dihydrocinnamic acid	0.03
methyl methoxy phenol	0.05	dimethoxy phenol	0.08	octanoic acid ethylcyclohexyl ester	0.03	dimethoxy propyl oxy benzaldehyde	0.03
thiophene ethyl isopentyl	0.05	ethylbenzenediol	0.97	dimethoxy phenol	0.46	methyl thiophenyl propanylacetate	0.12
methyl propyl thiophene	0.02	methyl dimethoxyphenol	0.84	dihydroxy acetophenone	0.05	dimethyl trihydroxy benzene	0.03
methoxyphenol	0.16	methoxybenzenediol	2.98	isopropoxy methoxyphenyl propanone	0.34	dihydoxy di(acetoxymethyl)benzene	0.09
dimethyl methoxy phenol	0.30	ethylbenzenediol	0.67	dimethoxypropenyl phenol	0.09		
dimethoxy dimethyl phenol	0.21	dimethyl benzenediol	0.24	dimethoxypropenyl phenol	0.05		
